# Microscale BTS sculptured by electron beam

**DOI:** 10.1007/s42649-019-0006-4

**Published:** 2019-04-29

**Authors:** Haneul Choi, Young Woo Jeong, Hye Jung Chang

**Affiliations:** 1 Advanced Analysis Center, Korea Institute of Science and Technology, Seoul, 02792 Republic of Korea; 2 Division of Nano & Information Technology, KIST School, University of Science and Technology, Seoul, 02792 Republic of Korea

**Keywords:** Electron microscope, Focused ion beam, Patterning

## Abstract

We applied the advanced bitmap-assisted patterning function of focused ion beam to fabricate microscale sculpture of the ‘BangTanSoNyeonDan’ known as BTS members, the world-wide famous K-pop boyband. With the help of an electron microscope, you can carve your idols on your accessories at micro scale. Fun applications of electron microscopes are not limited to science.

## Description

Focused Ion Beam (FIB) is a technique used for site-specific deposition and ablation of materials. In particular, Dual Beam Focused Ion Beam (Dual Beam FIB) which resembles a scanning electron microscope (SEM) setup incorporates electron gun and ion gun. Thus, it allows simultaneous patterning by ions and nanometer scale imaging by electron. Using the patterning function in the FIB software, we can fabricate complex structures employing user defined inputs such as direct import of graphical bitmap files as well as geometrical patterns like circle, rectangle and polygon (Zhou et al. 2005; Qian et al. 2008). This function has become an increasingly popular tool for the manufacturing various types of micro−/nanostructures and devices for different applications (Qian et al. 2008; Shang-En et al. 2007; Kuwabata et al. 2014; Wang et al. 2015). In this study, we applied the digital pattern generation functions to fabricate a microscale sculpture of a global K-pop boyband ‘BangTanSoNyeonDan’ known as BTS.

**Fig. 1:**
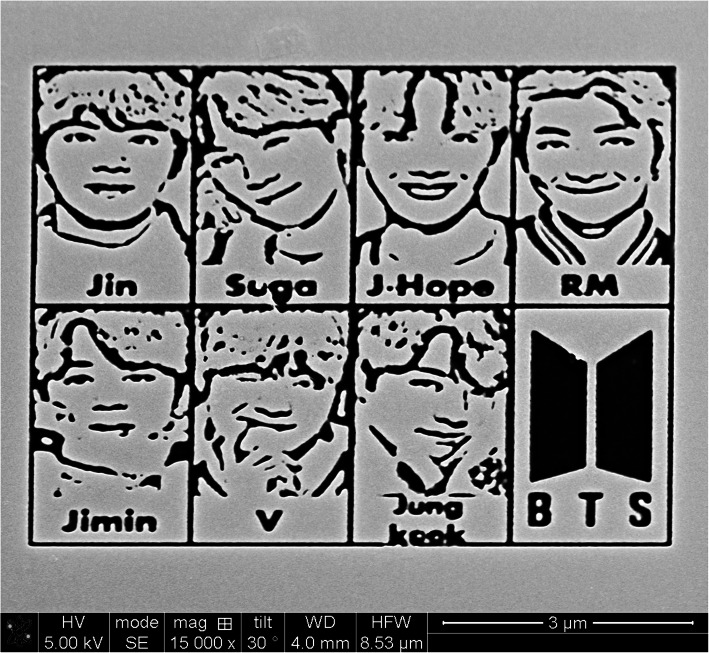
A SEM image of micro-scale BTS members' portrait patterned by bitmap-assisted patterning function of FIB

Ga^+^ ion etching on a Si (100) was carried out using Dual Beam FIB (Helios NanoLab 600, FEI). We converted the original colored photo image of BTS into bitmap file into gray scale. The bitmap file with 1000 pixels was imported into the FIB software. Pixels lying in the white regions were exposed to the ion milling. The Ga^+^ ion energy, beam current and dwell time are defined as 30 keV, 9.7 pA and 1 μs, respectively. And total ion dose was controlled by varying the irradiation time which was 35 s for the Figure 1.

Figure 1 is a sculptured portrait of BTS members; Jin, Suga, J-Hope, RM, Jimin, V and Jungkook. It is a world-wide famous boy band from South Korea (Wikipedia contributors 2019). In October 2018, Time Magazine featured BTS on the cover of their global edition, naming them “Next Generation Leaders”. On November 9, 2018 Love Yourself: Answer became the first Korean album to be certified Gold (500,000+ units), and BTS became the first Korean group to get a Platinum (1,000,000+ units) certification with the single ‘Mic Drop’ in the United States. Moreover, at the United Nations General Assembly on September 24, 2018, they received the attention of the world with the massage of “Love yourself and Find your voice by speaking yourself.”
